# Contribution to the taxonomy of Mexican Tersilochinae (Hymenoptera, Ichneumonidae), with descriptions of five new species

**DOI:** 10.3897/zookeys.974.54536

**Published:** 2020-10-07

**Authors:** Andrey I. Khalaim, Enrique Ruíz-Cancino

**Affiliations:** 1 Facultad de Ingeniería y Ciencias, Universidad Autónoma de Tamaulipas, Cd. Victoria, Mexico Universidad Autónoma de Tamaulipas Victoria Mexico; 2 Zoological Institute, Russian Academy of Sciences, St. Petersburg, Russia Russian Academy of Sciences St. Petersburg Russia

**Keywords:** Central America, keys, Mexico, new combination, new species, North America, Panama, parasitoids, taxonomy

## Abstract

Five new species of Tersilochinae (Ichneumonidae) are described from Mexico: *Meggoleus hidalgoensis***sp. nov.**, *M. whartoni* Khalaim, **sp. nov.**, *Phradis belovi* Khalaim, **sp. nov.**, *Stethantyx covida***sp. nov.**, and *St. oaxacana***sp. nov.***Meggoleus whartoni* Khalaim, **sp. nov.** is also recorded from Panama, and *St. covida***sp. nov.** from Guatemala. The species recently described from Mexico *Probles contrerasi* Khalaim & Ruíz-Cancino is transferred to the genus *Gelanes* Horstmann, **comb. nov.** A partial key to the species of *Meggoleus* with small propodeal spiracles and a key to Mexican species of *Phradis* are provided.

## Abstract (es)

Se describen cinco especies nuevas de Tersilochinae (Ichneumonidae) para México: *Meggoleus hidalgoensis***sp. nov.**, *M. whartoni* Khalaim, **sp. nov.**, *Phradis belovi* Khalaim, **sp. nov.**, *Stethantyx covida***sp. nov.** y *St. oaxacana***sp. nov.***Meggoleus whartoni* Khalaim, **sp. nov.** se registra también para Panamá y *St. covida***sp. nov.** para Guatemala. *Probles contrerasi*, una especie recientemente descrita para México, se transfiere al género *Gelanes* Horstmann, **comb. nov.** Se elaboró una clave parcial de las especies de *Meggoleus* con espiráculos propodeales pequeños y una clave para las especies mexicanas de *Phradis*.

## Introduction

The Tersilochinae is a moderately large, cosmopolitan subfamily of parasitoid wasps comprising more than 560 described species in 27 genera (Yu et al. 2016; Khalaim pers. obs.). The primary hosts of tersilochine parasitoids are larvae of various Coleoptera, but lepidopteran (Lepidoptera: Eriocraniidae) and symphytan (Hymenoptera: Tenthredinidae, Xyelidae) larvae also serve as hosts of some tersilochine taxa (Yu et al. 2016).

The Mexican fauna of Tersilochinae was virtually unknown until the 21^st^ century, being represented by only one species, *Stethantyx nearctica* Townes, 1971 recorded from northern Mexico. Due to our and several other researchers work, many Tersilochinae taxa have been described or recorded from Mexico since 2002 (see Yu et al. 2016; Khalaim and Ruíz-Cancino 2017, 2018, 2019), and currently ten genera with 29 species are known from Mexico: *Allophrys* Förster (1 species), *Aneuclis* Förster (2 species), *Barycnemis* Förster (2 species), *Diaparsis* Förster (1 species), *Gelanes* Horstmann (1 species), *Labilochus* Khalaim (1 species), *Meggoleus* Townes (1 species), *Phradis* (2 species), *Probles* Förster (12 species), and *Stethantyx* Townes (6 species). Despite the extensive study of Mexican Tersilochinae in the past two decades, many Mexican species still remain undescribed, and some genera require further investigation.

The aim of this work is to describe five new species in the genera *Meggoleus*, *Phradis*, and *Stethantyx* from Mexico, revise the generic positions of recently described species of *Probles*, and provide identification keys to the species of *Meggoleus* and *Phradis*.

## Materials and methods

A large number of tersilochine specimens was examined from the Universidad Autónoma de Tamaulipas, Cd. Victoria, Mexico (**UAT**); Instituto de Biología, Universidad Nacional Autónoma de México, D.F., Mexico (**UNAM**); and Texas A&M University, College Station, Texas, USA (**TAMU**). Additional paratype of *Stethantyx covida* sp. nov. was loaned from the University of California, Riverside, California, USA (**UCR**), and several paratypes are preserved in the Natural History Museum, London, UK (**BMNH**), Florida State Collection of Arthropods, Gainesville, Florida, USA (**FSCA**) and Zoological Institute of the Russian Academy of Sciences, St. Petersburg, Russia (**ZISP**).

Morphological terminology follows that of Townes (1969) with changes according to Khalaim (2011). Photographs were taken in the Zoological Institute RAS (St. Petersburg, Russia), with a Canon EOS 70D digital camera attached to an Olympus SZX10 stereomicroscope. Images were assembled with Helicon Focus 6 Pro software. General data on the distribution and biology of the genera follow the catalogue TaxaPad (Yu et al. 2016).

## Taxonomy

### Genus Gelanes

Taxon classificationAnimaliaHymenopteraIchneumonidae

Horstmann, 1981

015EF300-75AC-5476-AF48-73CC221DB427

#### Type species.

*Thersilochus fusculus* Holmgren, 1860.

A moderately large Holarctic genus with 15 species in the Nearctic region (including two species from Mexico) and 20 species in the Palaearctic region. Parasitoids of xyelid sawflies (Hymenoptera: Xyelidae: *Xyela* spp.) feeding in staminate cones on pines (Pinaceae: *Pinus* spp.) (Khalaim and Blank 2011).

Two species of *Gelanes* are known to occur in Mexico: *G. horstmanni* Khalaim from the State of Tlaxcala in Central Mexico (Khalaim and Ruíz-Cancino 2017) and *G. contrerasi* (Khalaim & Ruíz-Cancino), comb. nov. The latter species was recently described as a species of *Probles* Förster from the State of Hidalgo in Central Mexico (Khalaim and Ruíz-Cancino 2019).

### Gelanes contrerasi

Taxon classificationAnimaliaHymenopteraIchneumonidae

(Khalaim & Ruíz-Cancino, 2019) comb. nov.

86C1E5DC-399E-5BBC-AF2D-1004EECAC31D

Probles (Euporizon) contrerasi Khalaim & Ruíz-Cancino, 2019: 210 [holotype female (UNAM), Mexico, Hidalgo, Huasca de Ocampo, Rancho Santa Elena, 20°06'N, 98°31'W, 2330–2535 m, Hueyapan River, 13.VI.2010, coll. A. Contreras R. et al.].

#### Remarks.

This species was recently described in the genus *Probles* based on a single female from the State of Hidalgo in Central Mexico. The species has a slender first metasomal tergite with glymma situated slightly behind the middle, thin and long foveate groove of mesopleuron and long thyridial depression (see figs 20–25 in Khalaim and Ruíz-Cancino 2019: 211), and therefore formally it runs to *Probles*. However, we consider that its unusually broad clypeus with a flat area centrally and highly polished genae and mesopleuron better correspond with the genus *Gelanes* (comb. nov.).

*Gelanes contrerasi* may easily be distinguished from another Mexican species, *G. horstmanni* Khalaim, by its genae constricted behind eyes in dorsal view (swollen in *G. horstmanni*), slender antennal flagellum with 16 flagellomeres (robust, with 25 flagellomeres in *G. horstmanni*), and longer basal area of propodeum and second metasomal tergite. In the key to the Nearctic species of *Gelanes* (Horstmann 2013b: 238), *G. contrerasi* runs to *G. incisus* Horstmann and *G. punctipleuris* Horstmann in couplet 3, but differs from the both by having longer genae, propodeum with basal area very narrow and longer than the apical area, and longer second metasomal tergite.

### Genus Meggoleus

Taxon classificationAnimaliaHymenopteraIchneumonidae

Townes, 1971

8530B72A-9C24-52E4-9AED-C6EB386EEC07

#### Type species.

*Meggoleus spirator* Townes, 1971.

It is a small Neotropical genus with five species; one Afrotropical species with large propodeal spiracles described in the genus *Meggoleus* (Khalaim 2007) was recently found to belong to the genus *Allophrys* Förster (Khalaim 2017). Nothing is known about host range of any *Meggoleus* species.

Three species of *Meggoleus* occurring in South America were revised by Alvarado (2012). Two of them, *M. spirator* Townes and *M. pampahermosensis* Alvarado, are known from Costa Rica (Khalaim and Broad 2012, Khalaim et al. 2018), and the latter species was recorded also from Mexico (Khalaim et al. 2018). In this paper, we describe two new species of *Meggoleus* from Mexico and Panama. Both new species possess small propodeal spiracles, clearly differing from three previously known taxa which are characterized by strongly enlarged propodeal spiracles. Record of *M. hidalgoensis* sp. nov. from Tamaulipas is a northernmost known locality for the genus *Meggoleus*. A partial key to the species of *Meggoleus* with small propodeal spiracles is provided.

### Key to species of *Meggoleus* (partial)

**Table:** 

1	Propodeal spiracle small, not or very weakly enlarged (Figs 6, 12)	**2**
–	Propodeal spiracle strongly enlarged	**see key by Alvarado (2012)**
2	Antennal flagellum black but with two or three distal flagellomeres white (Fig. 10). Scutellum with lateral longitudinal carinae present at basal 0.1–0.2. Intercubitus (2rs-m) short and very thick, much shorter (0.5 × or less) than abscissa of M between 2rs-m and 2m-cu	***M. whartoni* sp. nov.**
–	Antennal flagellum entirely black (Fig. 3). Scutellum with lateral longitudinal carinae present at basal 0.3–0.5. Intercubitus (2rs-m) slightly thickened, ca. as long as abscissa of M between 2rs-m and 2m-cu	***M. hidalgoensis* sp. nov.**

### Meggoleus hidalgoensis

Taxon classificationAnimaliaHymenopteraIchneumonidae

Khalaim & Ruíz-Cancino sp. nov.

003457F1-8B95-5E09-9B93-662DFE6398D1

http://zoobank.org/1C8E9237-B9EC-47D4-9897-774F77C429B6

Figures 1–8

#### Differential diagnosis.

The new species differs from other species of *Meggoleus* by the combination of its relatively small, not enlarged propodeal spiracles (Figs 5, 6) and black antenna (Fig. 3).

#### Description.

**Female.** Body length 4.4 mm. Fore wing length 3.4 mm.

Head, in dorsal view, strongly constricted, almost straight (holotype) or rounded posterior to eyes; gena 0.65–0.75 × as long as eye width. Eyes glabrous. Clypeus relatively large, lenticular, ca. 2.6 × as broad as long (Fig. 4), weakly convex in lateral view, separated from face by sharp furrow; smooth, with scattered punctures in upper part, sometimes slightly scabrous near upper and lower margins. Mandible not constricted, with upper and lower margins mostly subparallel, distinctly widened at level of teeth; teeth somewhat divergent, upper tooth ca. 1.5 × longer than the lower. Malar space 0.9–1.2 × as long as basal mandibular width. Antennal flagellum (Fig. 3) with 15 or 16 flagellomeres, basally very slender; basal flagellomeres more than 2.5 × as long as broad, subapical flagellomeres distinctly elongate; flagellomeres 4–7 bearing long and thin subapical finger-shaped structures on outer surface (hardly discernible in light microscope). Face weakly convex. Face, frons, and vertex subpolished, weakly shining, with very fine, mostly indistinct punctures. Gena polished, with fine and sparse punctures. Occipital carina complete, somewhat dipped mediodorsally, evenly arcuate in dorsal view. Hypostomal carina absent at least in lower part.

**Figures 1–8.**
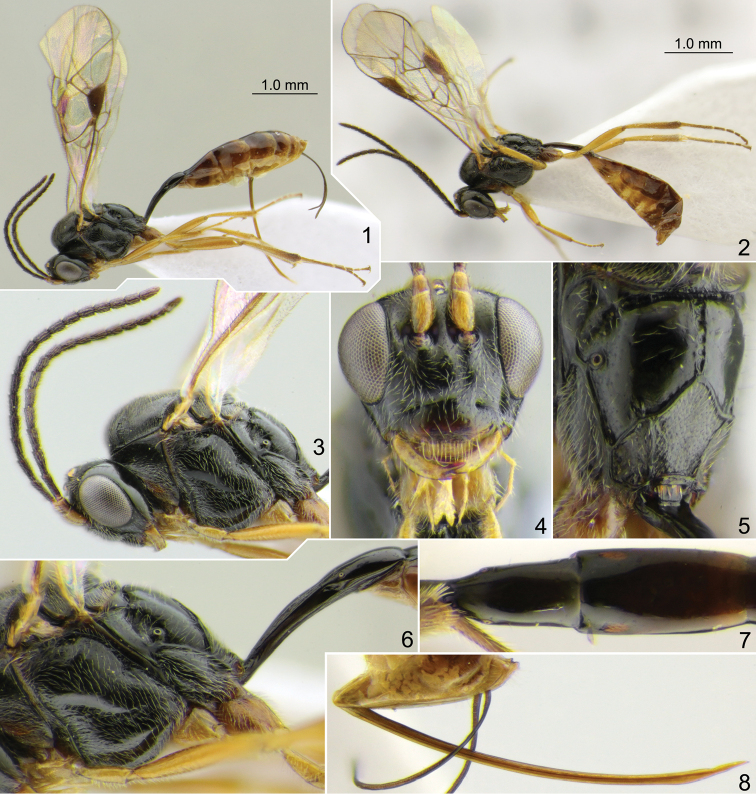
*Meggoleus hidalgoensis* sp. nov., holotype female (all except 2) and paratype male (2) **1, 2** habitus, lateral view **3** head with antennae and mesosoma, lateral view **4** head, front view **5** propodeum dorso-postero-lateral view **6** posterior part of mesosoma and base of metasoma, lateral view **7** postpetiole and second tergite, dorsal view **8** apex of metasoma with ovipositor, lateral view.

Mesoscutum very finely and shallowly granulate, sometimes almost smooth on lateral lobes, impunctate or with very fine inconspicuous punctures, weakly shining to dull. Notaulus impressed, with distinct wrinkle on anterolateral side of mesoscutum (Fig. 3). Scutellum with lateral longitudinal carinae at basal 0.3–0.5. Epicnemial carina not reaching front margin of mesopleuron, continuing above along front margin of mesopleuron towards subtegular ridge, and vanishing there (Fig. 6). Foveate groove long, narrow, sharp, anteriorly upcurved, with distinct transverse wrinkles (Figs 3, 6). Mesopleuron smooth, very finely punctate (sometimes punctures indistinct), with impunctate area centrally. Propodeal spiracle round, not enlarged, separated from pleural carina by 1.0–2.0 × diameter of spiracle (Figs 3, 5). Propodeum with narrow median longitudinal furrow which is 0.7–1.1 × as long as apical area (Fig. 5). Dorsolateral area polished, impunctate (Figs 5, 6). Apical area flat, rounded or pointed anteriorly (Fig. 5); apical longitudinal carinae usually complete and reaching transverse carina anteriorly, sometimes partly obliterated.

Fore wing with second recurrent vein (2m-cu) postfurcal, weakly pigmented in anterior part and distinct posteriorly. First abscissa of radius (Rs+2r) straight, somewhat longer than width of pterostigma. First and second abscissae of radius (Rs+2r and Rs) meeting at slightly acute angle. Intercubitus (2rs-m) slightly thickened, approximately as long as abscissa of cubitus between intercubitus and second recurrent vein (abscissa of M between 2rs-m and 2m-cu). Metacarpus (R1) almost reaching apex of fore wing. Second abscissa of postnervulus (Cu&2cu-a) present, thus brachial cell is closed posteriorly. Hind wing with nervellus (cu1&cu-a) weakly reclivous. Legs slender. Tarsal claws long and slender, not pectinate.

First tergite ca. 3.8 × as long as posteriorly broad, smooth, sometimes with very weak striae laterally just before glymma; petiole more or less trapeziform in cross-section centrally; in dorsal view, postpetiole distinctly widened at base, wider than petiole and clearly separated from it (Fig. 7); in lateral view, upper margin of tergite weakly arcuate in basal 0.6 and somewhat stronger arcuate in apical 0.4 (Fig. 6). Glymma distinct, situated in apical 0.6 of tergite, joining by weak groove with lower part of postpetiole (Fig. 6), but sometimes this groove is vestigial and glymma is virtually isolated. Second tergite ca. 1.85 × as long as anteriorly broad (Fig. 7). Thyridial depression shallow to deep, 2.0–3.0 × as long as broad, with posterior end rounded. Ovipositor weakly and nearly evenly bent upwards over its total length, with weak dorsal subapical depression (Fig. 8); sheath 1.0–1.4 × as long as first tergite (1.4 × in holotype).

Head and mesosoma black. Palpi, mandible (teeth dark red), lower 0.4–0.5 of clypeus and tegula brownish yellow. Scape and pedicel of antenna yellow-brown ventrally and brown dorsally; flagellum brownish black, sometimes pale at base. Pterostigma brown. Legs brownish yellow; hind coxa darkened with brown at base; apex of hind tibia and hind tarsus infuscate. First tergite brown to dark brown. Metasoma posterior to first tergite brown or dark brown dorsally to brownish yellow ventrally.

**Male.** Similar to female; flagellum slender, more or less tapered towards apex, with 16 flagellomeres (Fig. 2).

#### Variation.

Pale specimens have head and mesosoma mostly reddish brown rather than black, and metasoma pale brown to yellow. Two females from the State of Oaxaca possess foveate groove of mesopleuron very thin, represented by a line of sharp and deep pits.

#### Etymology.

The species is named after the type locality, [State of] Hidalgo.

#### Material examined.

***Holotype*** female (UNAM), Mexico, Hidalgo, Huasca de Ocampo, Rancho Santa Elena, 20°06'N, 98°31'W, 2330–2535 m, Hueyapan River, 25.I–23.II.2006, coll. A. Contreras R. et al.

***Paratypes*. Mexico**: 1 female (UAT, apices of antennae absent), **Tamaulipas**, Gómez Farías, La Gloria, 11.III.1995, coll. D. Zuñiga. 1 female (UAT, head absent), 1 male (UNAM), **Hidalgo**, same data as holotype, but 29.XI–26.XII.2005. 1 female, 1 male (ZISP), **Morelos**, N of Tepoztlán, path to El Tepozteco, 1800–2000 m, 11.X.2014, coll. A.I. Khalaim. 1 female (TAMU), **Veracruz**, 7 mi. W of Jalapa [Xalapa], 24–25.III.1974, coll. J.C. Schaffner. 1 female (TAMU), **Oaxaca**, 10.8 mi. (= 17.4 km) S of El Punto, 6100 ft (= 1860 m), 19.VII.1987, coll. R. Wharton. 1 female (TAMU), Oaxaca, 15 mi. (= 24 km) NE of Ixtlán de Juárez, Llano de las Flores, 21.VII.1985, coll. J.B. Woolley & G. Zolnerovich.

#### Distribution.

Mexico (Tamaulipas, Hidalgo, Morelos, Veracruz, Oaxaca).

### Meggoleus whartoni

Taxon classificationAnimaliaHymenopteraIchneumonidae

Khalaim sp. nov.

F4DBC6E5-8A16-514E-B302-3D62E26AF8E6

http://zoobank.org/489674A1-97D4-47FB-95D7-98CD4C7601F9

Figures 9–14

#### Differential diagnosis.

The new species is easily distinguished from all other species of *Meggoleus* by the flagellum with distal end white (Fig. 10). It is very similar to *M. hidalgoensis* sp. nov. as both have small propodeal spiracles but differs from this species, in addition to color pattern of the flagellum, by scutellum with shorter lateral longitudinal carinae and fore wing with short and thick intercubitus (2rs-m).

#### Description.

**Female.** Body length 3.7 mm. Fore wing length 2.8 mm.

Head, in dorsal view, strongly constricted, weakly rounded posterior to eyes; gena 0.6–0.65 × as long as eye width. Eyes glabrous. Clypeus relatively large, almost lenticular (slightly truncated ventrally), ca. 2.5 × as broad as long (Fig. 11), very weakly convex in lateral view, separated from face by fine furrow; smooth, with very fine punctures in upper 0.3–0.5. Mandible slender, not constricted, with upper and lower margins mostly subparallel; upper tooth almost twice longer than the lower. Malar space approximately as long as basal mandibular width. Antennal flagellum (Fig. 10) with 15 flagellomeres, basally very slender; basal flagellomeres almost 2.5 × as long as broad, subapical flagellomeres distinctly elongate; flagellomeres 4 to 6 bearing long and thin subapical finger-shaped structures on outer surface. Face weakly convex. Face and frons subpolished, weakly shining, with very fine punctures. Vertex polished, with very fine and sparse punctures. Gena polished, impunctate. Occipital carina complete, somewhat dipped mediodorsally, evenly arcuate in dorsal view. Hypostomal carina present in upper part, weak or completely obliterated in lower part.

**Figures 9–14.**
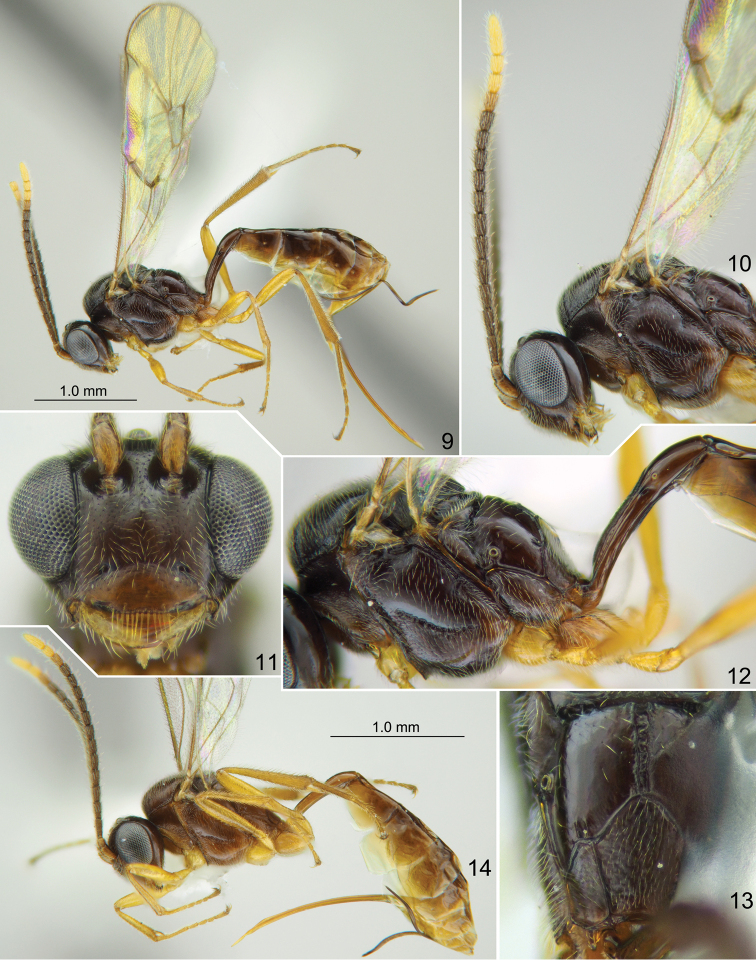
*Meggoleus whartoni* sp. nov., holotype (9–13) and paratype (14) females **9, 14** habitus lateral view **10** head with antennae and mesosoma, lateral view **11** head, front view **12** mesosoma and base of metasoma, lateral view **13** propodeum, dorsal view.

Mesoscutum granulate, impunctate, dull. Notaulus with distinct wrinkle on anterolateral side of mesoscutum (Fig. 10). Scutellum with lateral longitudinal carinae at basal 0.1–0.2. Epicnemial carina not reaching front margin of mesopleuron, continuing above along front margin of mesopleuron towards subtegular ridge, and vanishing there (Fig. 12). Foveate groove long, narrow and sharp, anteriorly upcurved, with distinct transverse wrinkles (Fig. 12). Mesopleuron smooth, with very fine inconspicuous punctures. Propodeal spiracle round, slightly enlarged, separated from pleural carina by 1.0–1.5 × diameter of spiracle (Fig. 12). Propodeum with narrow median longitudinal furrow which is more or less enclosed laterally by a pair of longitudinal carinae, and ca. 0.8 × as long as apical area (Fig. 13). Dorsolateral area polished, impunctate (Fig. 13). Apical area flat, anteriorly rounded (Fig. 13); apical longitudinal carinae complete, reaching transverse carina anteriorly.

Fore wing with second recurrent vein (2m-cu) postfurcal, weakly pigmented in anterior part and distinct posteriorly. First abscissa of radius (Rs+2r) straight, longer than width of pterostigma. First and second abscissae of radius (Rs+2r and Rs) meeting at slightly acute angle. Intercubitus (2rs-m) short and very thick, much shorter (0.5 × or less) than abscissa of cubitus between intercubitus and second recurrent vein (abscissa of M between 2rs-m and 2m-cu). Metacarpus (R1) almost reaching apex of fore wing. Second abscissa of postnervulus (Cu&2cu-a) present, thus brachial cell is closed posteriorly. Hind wing with nervellus (cu1&cu-a) weakly reclivous. Legs slender. Tarsal claws long and slender, not pectinate.

First tergite ca. 4.4 × as long as posteriorly broad, predominantly smooth, with weak striae laterally before glymma; petiole trapeziform in cross-section centrally; in dorsal view, postpetiole widened at base, distinctly broader than petiole and clearly separated from it; in lateral view, upper margin of tergite weakly arcuate in basal 0.6 and somewhat stronger arcuate in apical 0.4. Glymma distinct, situated in apical 0.6 of tergite, joining by weak (sometimes vestigial) groove with lower part of postpetiole (Fig. 12). Second tergite approximately twice as long as anteriorly broad. Thyridial depression deep, ca. 2.5 × as long as broad, with posterior end rounded. Ovipositor weakly and nearly evenly bent upwards over its total length, with weak dorsal subapical depression; sheath approximately as long as first tergite.

Head, mesosoma and first tergite of metasoma orange-brown to dark reddish brown. Palpi and mandible (teeth red) yellow. Clypeus yellow-brown or reddish brown, unicolorous or slightly darkened in upper part. Tegula brownish yellow. Scape and pedicel of antenna yellow-brown ventrally and brown dorsally; flagellum brownish black with two or three distal flagellomeres white (Fig. 10). Pterostigma brown. Legs brownish yellow, hind coxa slightly brownish at base. Metasoma posterior to first tergite predominantly brown, yellow ventrally.

**Male.** Unknown.

#### Etymology.

The species is named in honor of the American entomologist, expert in Braconidae and Ichneumonidae, Robert Wharton.

#### Material examined.

Holotype female (TAMU), Mexico, “Chiapas”.

Paratypes. **Panama**: 2 females (TAMU), Chiriquí Prov., National Park Volcan Baru, 3 km E of Cerro Punta, 08°50'55"N, 82°32'36"W, 7060 ft. (= 2155 m), 31.VII–4.VIII.1999, coll. A. Gillogly & J. Woolley, Malaise trap, 99/072.

#### Distribution.

South Mexico (Chiapas), Panama.

### Genus Phradis

Taxon classificationAnimaliaHymenopteraIchneumonidae

Förster, 1869

AD98DD68-DF6A-5B85-9D1B-B131282F4D8B

#### Type species.

Thersilochus (Phradis) brevis Brischke, 1880.

A moderately large predominantly Holarctic genus with 20 species in the Nearctic region (including two species from Mexico), ca. 40 species in the Palaearctic region, and several species known from Peru, South Africa, and Australia. In Europe, species of *Phradis* have been reared from sap beetle larvae (Coleoptera: Nitidulidae: *Meligethes* spp.) feeding on rape, but no host record is known for any Nearctic species (Horstmann 2013a).

Two species of *Phradis* were known from Mexico until now (Khalaim and Ruíz-Cancino 2018), and the third species, *P. belovi* sp. nov., is described from North Mexico in this paper. The genus is extremely rare in Mexico as all known Mexican species are represented by a single holotype. Horstmann (2013a) in his revision of the Nearctic fauna, also noted that many Nearctic species are rarely collected, and six of 18 revised species (33%) are known from only one specimen. A key to three Mexican species of *Phradis* is provided below.

### Key to species of *Phradis* occurring in Mexico

**Table:** 

1	Flagellum with 17 flagellomeres (Fig. 16). Fore wing with vein 2m-cu postfurcal. Notaulus very shallow, with short wrinkle or tubercle distant from anterolateral margin of mesoscutum. Propodeum mediodorsally with narrow longitudinal furrow, without delimited basal area; dorsolateral areas polished. Hind femur brownish yellow, not darkened (Fig. 15). Ovipositor sheath twice as long as first tergite. Hind femur brownish yellow	***P. belovi* sp. nov.**
–	Flagellum with 14 flagellomeres. Fore wing with vein 2m-cu interstitial. Notaulus with strong wrinkle on anterolateral side of mesoscutum. Propodeum mediodorsally with clearly delimited broad basal area; dorsolateral areas granulate. Ovipositor sheath 1.1–1.4 × as long as first tergite. Hind femur dark brown to black	**2**
2	Second flagellomere 2.5 × as long as broad. Apical area of propodeum flat. Second metasomal tergite 2.8 × as long as anteriorly broad. Ovipositor with apex needle-shaped, without dorsal notch; sheath 1.1 × as long as first tergite	***P. bufalosus* Khalaim & Ruíz-Cancino**
–	Second flagellomere 3.5 × as long as broad. Apical area of propodeum impressed along midline. Second metasomal tergite 3.6 × as long as anteriorly broad. Ovipositor evenly tapered apically, with weak but distinct dorsal subapical notch; sheath 1.4 × as long as first tergite	***P. nanacamilpus* Khalaim & Ruíz-Cancino**

### Phradis belovi

Taxon classificationAnimaliaHymenopteraIchneumonidae

Khalaim sp. nov.

53667187-01A0-5CEF-9407-79DE179EBE9E

http://zoobank.org/08038E88-9796-418C-9758-111496BA7E0A

Figures 15–20

#### Differential diagnosis.

**Figures 15–20.**
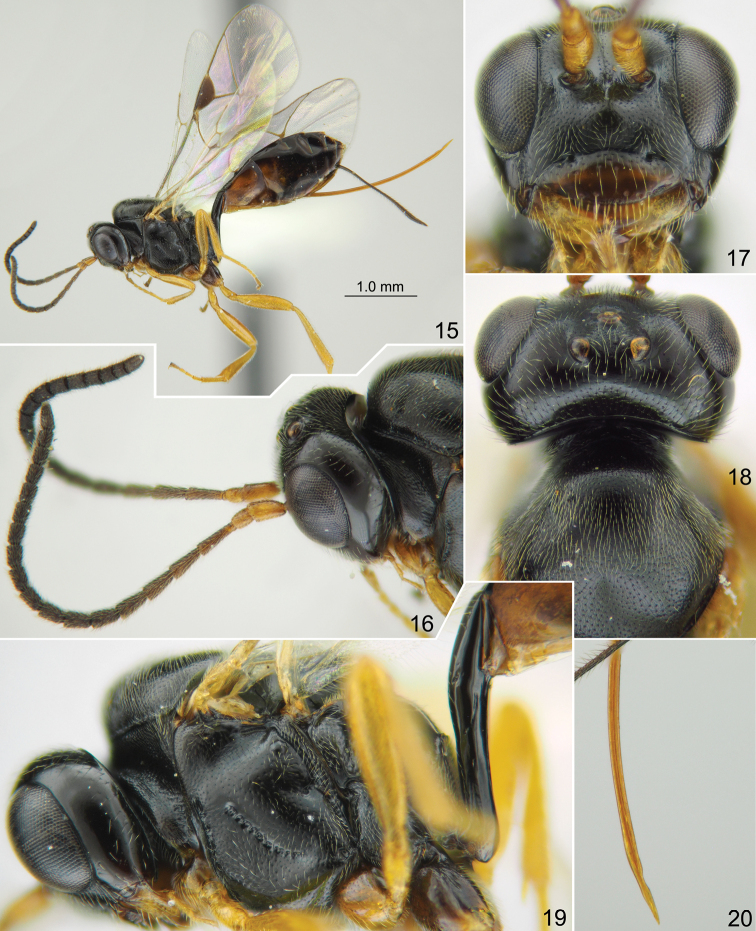
*Phradis belovi* sp. nov., holotype female **15** habitus, lateral view **16** head with antennae lateral view **17** head, front view **18** head and mesoscutum, dorsal view **19** head, mesosoma and base of metasoma, lateral view **20** apex of ovipositor, lateral view.

*Phradis belovi* sp. nov. may easily be recognized by the postfurcal second recurrent vein (2m-cu) in the fore wing, narrow and sharp foveate groove on the mesopleuron (Fig. 19), and propodeum with longitudinal furrow mediodorsally and polished dorsolateral areas. It differs from two other Mexican species of *Phradis* by features given in the key above. In the key to Nearctic species of *Phradis* (Horstmann 2013a: 68), *P. belovi* sp. nov. runs to *P. nitidipleuris* Horstmann in couplet 17, but differs from this species by the propodeum mediodorsally with longitudinal furrow (short and broad, irregularly wrinkled basal area in *P. nitidipleuris*, see fig. 77 in Horstmann 2013a: 82), longer metacarpus in the fore wing (Fig. 15 and Khalaim 2019: 413, fig. 52), and its narrow and sharp foveate groove on the mesopleuron (Fig. 19) (broad, with irregular wrinkles in *P. nitidipleuris*, see Khalaim 2019: 413, fig. 5).

#### Description.

**Female.** Body length 4.3 mm. Fore wing length 3.3 mm.

Head, in dorsal view, rounded posterior to eyes (Fig. 18); gena 0.65 × as long as eye width. Eyes glabrous. Clypeus relatively large, lenticular, 2.6 × as broad as long (Fig. 17), weakly convex in lateral view, separated from face by thin and sharp furrow, with flattened area in lower part centrally; smooth, with fine punctures on slightly scabrous background in upper 0.4. Mandible robust, not constricted (i.e. with upper and lower margins subparallel in front view); upper tooth somewhat longer than the lower. Malar space approximately as long as basal mandibular width. Antennal flagellum filiform, with 17 flagellomeres (Fig. 16); flagellomeres 2 and 3 ca. 2.5 × as long as broad, subapical flagellomeres slightly elongate; flagellomeres 4–7 bearing long and thin subapical finger-shaped structures on outer surface (hardly discernible in light microscope). Face with slightly elongated convexity centrally. Face, frons, and vertex with very fine and dense punctures; gena impunctate anteriorly, with fine and sparse punctures in posterior half. Face and frons subpolished, weakly shining. Vertex and gena polished. Occipital carina complete, weakly and evenly arcuate in dorsal view (Fig. 18), somewhat flattened mediodorsally.

Mesoscutum very finely and densely punctate on very finely and shallowly granulate background, weakly shining, except for central lobe which is dull, with somewhat denser granulation and mostly without distinct punctures. Notaulus very shallow, with short wrinkle or tubercle distant from anterolateral margin of mesoscutum. Scutellum with lateral longitudinal carinae at basal 0.1. Epicnemial carina with upper end at level of centre of pronotum, not reaching front margin of mesopleuron (Fig. 19). Foveate groove situated in center of mesopleuron, very narrow and sharp, anteriorly upcurved (Fig. 19). Mesopleuron smooth and shining, finely punctate. Propodeal spiracle small, round, separated from pleural carina by 1.5 × diameter of spiracle. Propodeum with dorsal part convex in lateral view (Fig. 19), with narrow median longitudinal furrow which is ca. 0.7 × as long as apical area. Dorsolateral area polished, impunctate. Apical area flat, widely rounded anteriorly; apical longitudinal carinae almost reaching transverse carina anteriorly, indistinct next to transverse carina because of irregular wrinkles.

Fore wing with second recurrent vein (2m-cu) distinctly postfurcal, weakly pigmented in anterior 0.6. First abscissa of radius (Rs+2r) straight, distinctly longer than width of pterostigma. First and second abscissae of radius (Rs+2r and Rs) meeting at right angle. Intercubitus (2rs-m) slightly thickened, twice as long as abscissa of cubitus between intercubitus and second recurrent vein (abscissa of M between 2rs-m and 2m-cu). Metacarpus (R1) reaching ca. 0.7 the distance from distal corner of radial cell to the tip of wing. Second abscissa of postnervulus (Cu&2cu-a) present but short, thus brachial cell is partly open posteriorly. Hind wing with nervellus (cu1&cu-a) straight, weakly reclivous. Legs slender. Tarsal claws slender, not pectinate.

First tergite ca. 4.1 × as long as posteriorly broad, smooth, without glymma but with sharp oblique groove (Fig. 19), with upper margin in lateral view straight in basal half and arcuate in apical half; petiole round in cross-section centrally. First tergite, in dorsal view, weakly and rather evenly widened from base towards apex, thus postpetiole is weakly separated from petiole. Second tergite 2.5 × as long as anteriorly broad. Thyridial depression distinct, ca. 2.5 × as long as broad, with posterior end somewhat pointed. Ovipositor weakly and evenly bent upwards over its total length, with weak dorsal subapical depression (Fig. 20); sheath twice as long as first tergite.

Head, mesosoma and first tergite of metasoma black; clypeus brown in lower 0.4 and dark brown in upper 0.4, with narrow transverse brownish yellow band. Palpi, mandible (teeth dark red) and tegula brownish yellow. Scape and pedicel of antenna yellow-brown, flagellum pale brown basally to black apically. Pterostigma brown. Legs brownish yellow; fore and mid coxae browish, hind coxa dark brown. Metasoma posterior to first tergite predominantly dark brown, tergites 2 and 3 laterally brown (Fig. 15).

**Male.** Unknown.

#### Etymology.

The species is named after my friend, the well-known entomologist Vassili Belov (TAMU).

#### Material examined.

Holotype female (TAMU), Mexico, Nuevo León, “3 mi. south Pacheco”, taken at light, 3.VII.1974, coll. Clark, Murraw, Asche & Schaffner.

#### Distribution.

Northeast Mexico (Nuevo León).

### Genus Stethantyx

Taxon classificationAnimaliaHymenopteraIchneumonidae

Townes, 1971

358F14B1-2C25-5220-8307-B620D473342D

#### Type species.

*Stethantyx nearctica* Townes, 1971.

Large and almost exclusively Neotropical genus with ca. 50 described and many undescribed species. Three species of *Stethantyx* occur in America north of Mexico, including one species introduced from South America (Horstmann 2010), and six Mexican species were reviewed by Khalaim and Ruíz-Cancino (2013). Species of the genus are known as parasitoids of coleopteran hosts of the families Curculionidae and Nitidulidae.

Two new species of *Stethantyx* are described here from Mexico, raising the total number of known *Stethantyx* species in Mexico to eight. The both new species possess right-angled radial cell in the fore wing and belong to the species group *radiata* (see Khalaim et al. 2015), while other six Mexican species belong to the species group *nearctica*, as they have abscissae of radius (Rs+2r and Rs) meeting at obtuse angle (see Khalaim and Broad 2013, Khalaim et al. 2013).

### Stethantyx covida

Taxon classificationAnimaliaHymenopteraIchneumonidae

Khalaim & Ruíz-Cancino sp. nov.

8029C501-BEFE-5443-BFE2-60CFA7CAA8BF

http://zoobank.org/FCCA3517-3788-403B-9FC9-4ED17AF839EF

Figures 21–26

#### Differential diagnosis.

**Figures 21–26.**
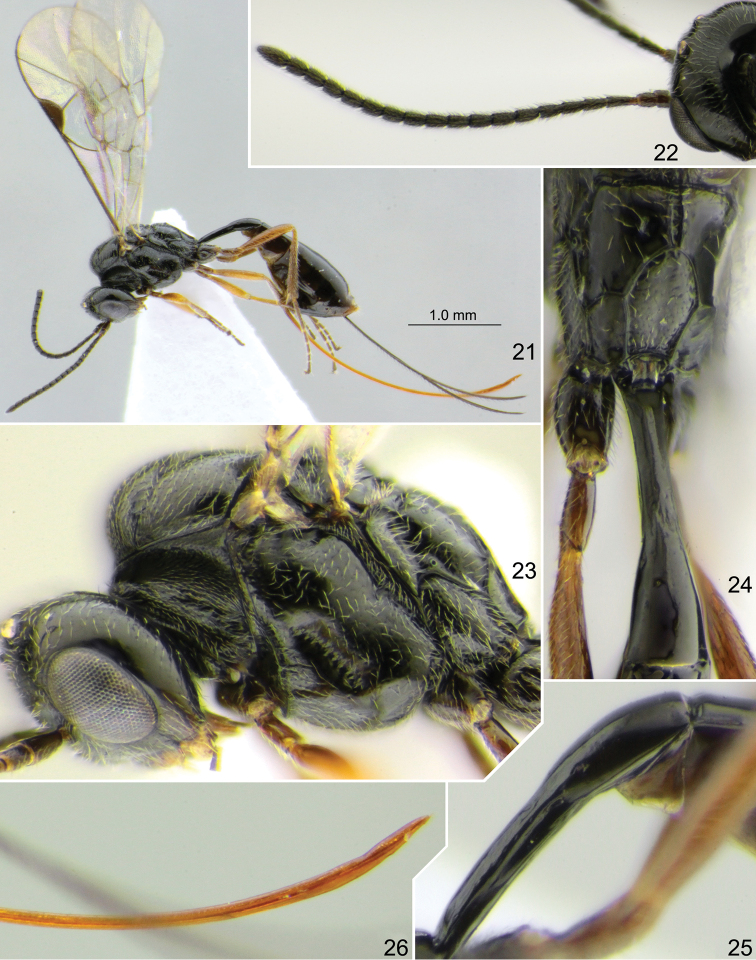
*Stethantyx covida* sp. nov., holotype female **21** habitus, lateral view **22** head with antennae, dorso-posterior view **23** head and mesosoma, lateral view **24** propodeum and first tergite, dorso-posterior view **25** first tergite, lateral view **26** apex of ovipositor, lateral view.

The new species differs from other species of *Stethantyx* with a right-angled first and second abscissae of radius (Rs+2r and Rs) by the combination of highly polished head and mesosoma, sharp and strongly oblique foveate groove of mesopleuron (Fig. 23), propodeum with narrow basal area (Fig. 24), and very long and slender ovipositor (Fig. 21). It is very similar to *St. oaxacana* sp. nov., but differs from this species by the shape of the ovipositor (Fig. 26), and somewhat longer gena, thyridial depression and second tergite.

#### Description.

**Female.** Body length 3.5 mm. Fore wing length 2.8 mm.

Head, in dorsal view, roundly constricted posterior to eyes; gena 0.9–1.0 × as long as eye width. Eyes glabrous. Clypeus lenticular (sometimes with lower margin slightly truncate), 3.2–3.4 × as broad as long, weakly convex in lateral view, with weak transverse ridge in lower 0.3–0.4, separated from face by sharp furrow; smooth, with fine punctures in upper part. Mandible slender, distinctly constricted in basal 0.3–0.4; upper tooth 2.0–2.5 × as long as the lower. Malar space 0.9–1.1 × as long as basal mandibular width. Antennal flagellum (Fig. 22) with 15–18 flagellomeres, filiform; flagellomeres 2–4 ca. 1.4–1.8 ×, subapical flagellomeres 1.1–1.3 × as long as broad; flagellomeres 4 to 6 bearing subapical finger-shaped structures on outer surface (hardly discernible in light microscope). Face weakly convex. Face, frons, and vertex subpolished; face and frons with very fine (sometimes indistinct) punctures. Gena polished, impunctate, or with very fine punctures in posterior part (near occipital carica). Occipital carina complete, evenly arcuate in dorsal view. Hypostomal carina present, complete.

Mesoscutum and mesopleuron very finely (sometimes indistinctly) punctate on smooth background; dorsolateral area of propodeum polished, impunctate. Notaulus with strong wrinkle on anterolateral side of mesoscutum. Scutellum with lateral longitudinal carinae at basal 0.3–0.5. Epicnemial carina not reaching front margin of mesopleuron, continuing above along front margin of mesopleuron, and vanishing there (Fig. 23). Foveate groove situated in anterior half of mesopleuron, deep, strongly oblique, almost straight, with distinct transverse wrinkles (Fig. 23). Propodeal spiracle small, adjacent to pleural carina or separated from it by one diameter of spiracle (Fig. 23). Propodeum with long and narrow basal area (basal longitudinal carinae parallel or weakly divergent anteriorly) which is 0.5–0.8 × (0.6 in holotype) as long as apical area (Fig. 24). Apical area flat, rounded to slightly pointed anteriorly; apical longitudinal carinae complete and reaching transverse carina anteriorly.

Fore wing with second recurrent vein (2m-cu) postfurcal, weakly pigmented in anterior part and distinct posteriorly. First abscissa of radius (Rs+2r) straight, longer than width of pterostigma. First and second abscissae of radius (Rs+2r and Rs) meeting at right or slightly acute angle. Intercubitus (2rs-m) slightly thickened, relatively long, distinctly longer than abscissa of cubitus between intercubitus and second recurrent vein (abscissa of M between 2rs-m and 2m-cu). Metacarpus (R1) short, not reaching apex of fore wing (Fig. 21). Second abscissa of postnervulus (Cu&2cu-a) present, thus brachial cell is closed posteriorly. Hind wing with nervellus (cu1&cu-a) weakly reclivous. Legs slender. Tarsal claws not pectinate.

First tergite 4.0 × as long as posteriorly broad, smooth, usually with longitudinal striae laterally before glymma and dorsally at apex of petiole; petiole slightly trapeziform in cross-section centrally; in dorsal view, postpetiole distinctly widened at base, wider than petiole and clearly separated from it; in lateral view, upper margin of tergite straight or weakly arcuate in basal 0.6–0.7 and arcuate in apical 0.3–0.4. Glymma (Fig. 25) small but distinct, situated in apical 0.55 of tergite and joining by fine groove (sometimes indistinct in small specimens) with lower part of postpetiole. Second tergite ca. 1.7 × as long as anteriorly broad. Thyridial depression deep, 2.0–3.0 × as long as broad, with posterior end usually rounded. Ovipositor bent upwards over its total length, with weak dorsal subapical depression and without teeth ventrally (Fig. 26); sheath 2.7–3.5 × (3.2 in holotype) as long as first tergite.

Head, mesosoma and first tergite brownish black to black; clypeus brownish yellow in lower 0.4 and dark brown in upper part, but sometimes clypeus is more or less entirely brownish yellow. Palpi and mandible (teeth red) brownish yellow. Tegula brownish yellow to brown. Antenna dark brown to black, scape and pedicel sometimes yellow-brown ventrally. Pterostigma brown. Legs brownish yellow; coxae and trochanters sometimes strongly darkened with brown (to almost black), tibiae and tarsi sometimes weakly to strongly infuscate (Fig. 21). Metasoma entirely or predominantly dark brown, sometimes brown posteriorly and ventrally.

**Male.** Similar to female but malar space somewhat shorter than basal mandibular width; basal area of propodeum very narrow and usually longer; and second metasomal tergite and thyridial depression longer.

#### Variation.

Two females from Nevado de Toluca (State of Mexico) possess second tergite 1.5–1.6 × as long as anteriorly broad. Epicnemial carina sometimes almost reaching front margin of mesopleuron. Foveate groove in small specimens sometimes weak.

#### Etymology.

This abundant Mexican species is named after the Covid-19 (Coronavirus) because the taxon was described while the outbreak of this virus in Mexico.

#### Material examined.

***Holotype*** female (UAT), Mexico, Tamaulipas, 6 km NE of Miquihuana, 23°36.125'N, 99°42.45'W, 2200–2600 m, 24.X.2008, coll. A.I. Khalaim.

***Paratypes*. Mexico**: 5 females (2 in BMNH, 1 in UAT, 2 in ZISP), same data as holotype. 12 females, 1 male (10 females in UAT; 2 females, 1 male in ZISP), **Tamaulipas**, [NE of] Miquihuana, Km. 15 [of road] from La Peña, 2500 m, pine forest, 16.VIII.2000, coll. D.R. Kasparyan. 14 females (1 in BMNH, 1 in FSCA, 12 in UAT), Tamaulipas, [NE of] Miquihuana, Km. 21 [of road] La Peña – Joya, pine forest, 16.IX.2000, coll. C. Covarrubias Dimas. 1 female (UAT), Tamaulipas, [NE of] Miquihuana, Km. 13 [of road] Aserradero – La Peña, herbs, 28.VIII.1993, coll. E. Ruíz-Cancino. 1 female (UAT), Tamaulipas, [NE of] Miquihuana, Km. 18 [of road] La Peña – Aserradero, pine forest, herbs, 24.X.2008, coll. E. Ruíz-Cancino. 1 female (ZISP), **Hidalgo**, 8 km N of Pachuca de Soto, National Park El Chico, 20°11.4'N, 98°44.55'W, 2950–3000 m, 27.III.2014, coll. A.I. Khalaim. 2 females (BMNH, FSCA), Hidalgo, 8 km N of Pachuca de Soto, National Park El Chico, 20°11.4'N, 98°44.55'W, 2800–2900 m, 18–22.XII.2014, coll. A.I. Khalaim. 2 females, 1 male (1 female, 1 male in UAT; 1 female in ZISP), **Tlaxcala**, 15 km SSE of Apizaco, north slope of La Malinche volcano, 19°16.97'N, 98°02.52'W, 3300–3800 m, 2.IV.2016, coll. A.I. Khalaim. 7 males (1 in BMNH, 1 in FSCA, 4 in UAT, 1 in ZISP), Tlaxcala, 15 km SSE of Apizaco, north slope of La Malinche volcano, 2550–3000 m, 1–2.X.2016, coll. A.I. Khalaim & A.E. Humala. 2 females, 1 male (UAT), **Mexico** [State of], NW slope of Nevado de Toluca volcano, 3150–3830 m, 29.IX.2016, coll. A.I. Khalaim. 1 female, 1 male (ZISP), **Morelos**, N of Tepoztlán, path to El Tepozteco, 1800–2000 m, 11.X.2014, coll. A.I. Khalaim. 2 males (TAMU), **Guerrero**, 7 mi. SW of Filo de Caballo, 12.VII.1985, coll. J. Woolley & G. Zolnerowich. 1 female (TAMU), **Oaxaca**, Llano de las Flores, 8900 ft (= 2715 m), 17–19.VII.1987, coll. R. Wharton. 2 females (TAMU), Oaxaca, 15 mi. (= 24 km) NE of Ixtlán de Juárez, Llano de las Flores, 21.VII.1985, coll. J.B. Woolley & G. Zolnerovich. 1 female (TAMU), Oaxaca, 10.8 mi. (= 17.4 km) S of El Punto, 6100 ft (= 1860 m), 19.VII.1987, coll. R. Wharton. 1 female (UNAM), Oaxaca, Santiago Comaltepec, 17.58429N, 96.49398W, 2332 m, 6.VI.2009, coll. H. Clebsch & A. Zaldívar. 1 female (UAT), 1 male (UNAM), same data but 29.XI–26.XII.2005 (head in female absent). 6 females, 12 males (5 females, 11 males in UNAM; 1 female, 1 male in UAT), Oaxaca, Santiago Comaltepec, 17.58424N, 96.49428W, 2427 m, humid oak-pine forest, Malaise trap, 12–20.VI.2007, coll. H. Clebsch. **Guatemala**: 1 female (UCR), Sacatepéquez [Department], Sumpango, Durwest Farm, 14°40'17"N, 90°43'11"W, 3–10.II.2007, coll. M. Hoddle.

#### Distribution.

Northeast, central, and south Mexico (Tamaulipas, Hidalgo, Tlaxcala, Mexico, Guerrero, Oaxaca), Guatemala.

### Stethantyx oaxacana

Taxon classificationAnimaliaHymenopteraIchneumonidae

Khalaim & Ruíz-Cancino sp. nov.

2BF4E5B2-A4CC-5008-AD10-5BB9ED830524

http://zoobank.org/56BC0231-F72C-4D74-B9AC-DBBEC699674D

Figures 27–32

#### Differential diagnosis.

The new species is very similar to *St. covida* sp. nov. but differs from this species in the shape of the ovipositor (Fig. 32), and shorter gena and second tergite. *Stethantyx oaxacana* sp. nov. also resembles *St. radiata* Khalaim & Sääksjärvi as both have similar shape of the ovipositor apex, but distinct in having clypeus separated from face by sharp furrow, less punctate head and mesosoma, and longer ovipositor.

#### Description.

**Female.** Body length 3.4 mm. Fore wing length 2.7 mm.

Head, in dorsal view, roundly constricted posterior to eyes (Fig. 28); gena ca. 0.7 × as long as eye width. Eyes glabrous. Clypeus lenticular, ca. 2.8 × as broad as long, weakly convex in lateral view, separated from face by sharp furrow; smooth, with fine scattered punctures in upper part. Mandible slender, weakly constricted in basal half; upper tooth twice longer than the lower. Malar space 1.0–1.1 × as long as basal mandibular width. Antennal flagellum (Fig. 29) with 16–17 flagellomeres, filiform; subbasal flagellomeres 1.5–1.8 ×, subapical flagellomeres 1.2–1.3 × as long as broad; flagellomeres 4 to 6 bearing subapical finger-shaped structures on outer surface (hardly discernible in light microscope). Face weakly convex. Face and frons finely (sometimes indistinctly) punctate on smooth or slightly scabrous background. Vertex and gena polished, without distinct punctures. Occipital carina complete, evenly arcuate in dorsal view. Hypostomal carina present, complete.

**Figures 27–32.**
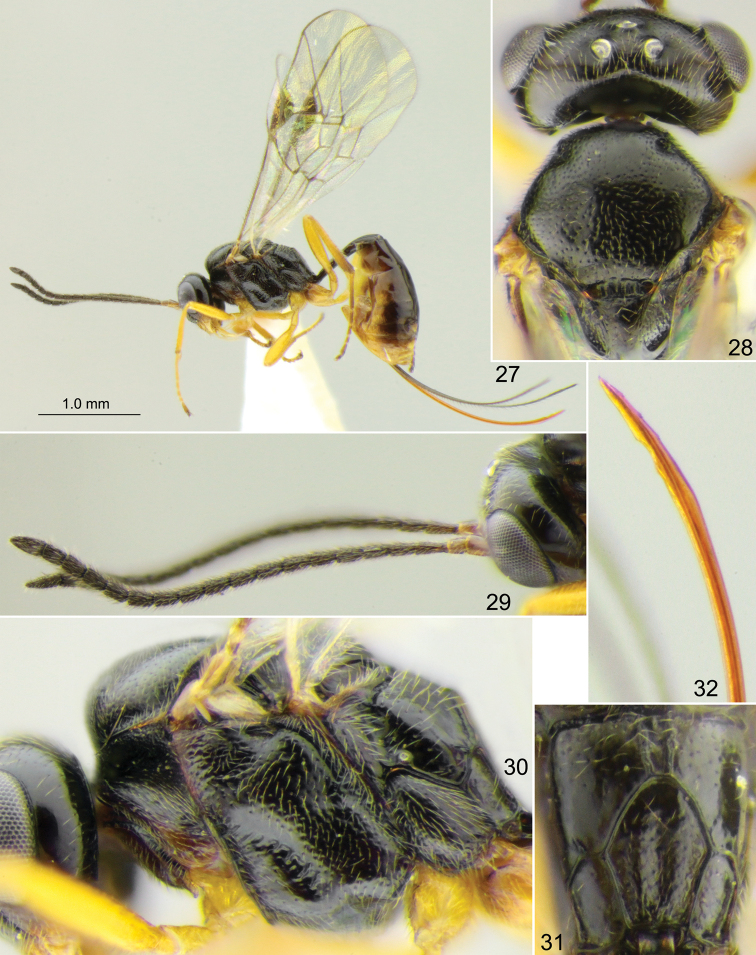
*Stethantyx oaxacana* sp. nov., holotype female **27** habitus, lateral view **28** head and mesoscutum, dorsal view **29** head with antennae, lateral view **30** head and mesosoma, lateral view **31** propodeum, dorsal view **32** apex of ovipositor, lateral view.

Mesoscutum and mesopleuron finely punctate on smooth background. Notaulus with strong wrinkle on anterolateral side of mesoscutum. Scutellum with lateral longitudinal carinae at basal 0.3–0.5. Epicnemial carina not reaching front margin of mesopleuron, continuing above along front margin of mesopleuron and vanishing there (Fig. 30). Foveate groove situated in anterior half of mesopleuron, deep, strongly oblique, almost straight, with distinct transverse wrinkles (Fig. 30). Propodeal spiracle adjacent to pleural carina or separated from it by less than one diameter of spiracle (Fig. 30). Propodeum with rectangular or slightly widened anteriorly basal area which is 2.0–4.0 × (2.0 in holotype) as long as broad and 0.4–0.8 × (0.4 in holotype) as long as apical area (Fig. 31). Dorsolateral area polished, with fine punctures in holotype (Fig. 31) and impunctate in paratypes. Apical area flat, rounded anteriorly (Fig. 31); apical longitudinal carinae complete and reaching transverse carina anteriorly.

Fore wing with second recurrent vein (2m-cu) postfurcal, weakly pigmented in anterior part and distinct posteriorly. First abscissa of radius (Rs+2r) straight, longer than width of pterostigma. First and second abscissae of radius (Rs+2r and Rs) meeting at slightly acute angle. Intercubitus (2rs-m) slightly thickened, approximately twice longer than abscissa of cubitus between intercubitus and second recurrent vein (abscissa of M between 2rs-m and 2m-cu). Metacarpus (R1) not reaching apex of fore wing. Second abscissa of postnervulus (Cu&2cu-a) present, thus brachial cell is closed posteriorly. Hind wing with nervellus (cu1&cu-a) weakly reclivous. Legs slender. Tarsal claws not pectinate.

First tergite 3.7 × as long as posteriorly broad, smooth, sometimes with longitudinal striae laterally before glymma and dorsally at apex of petiole; petiole rounded or slightly trapeziform in cross-section centrally; in dorsal view, postpetiole distinctly widened at base, wider than petiole and clearly separated from it; in lateral view, upper margin of tergite weakly arcuate in basal 0.6 and stronger arcuate in apical 0.4. Glymma small but distinct, situated in apical 0.55 of tergite and joining by fine groove with lower part of postpetiole. Second tergite ca. 1.25 × as long as anteriorly broad. Thyridial depression deep, ca. 1.5 × as long as broad, with posterior end rounded. Ovipositor bent upwards over its total length, with two dorsal subapical teeth and approximately three very small teeth ventrally (Fig. 32); sheath 2.3–2.6 × as long as first tergite (2.6 × in holotype).

Head and mesosoma predominantly brown to dark reddish brown (paratypes) or more or less entirely black (holotype); lower part of gena (near mandible) yellowish. Palpi and mandible (teeth red) yellow. Clypeus yellow, sometimes brownish in upper part. Tegula yellow or brownish yellow. Scape and pedicel of antenna yellowish brown; flagellum brownish black. Pterostigma brown. Legs brownish yellow; hind coxa sometimes darkened with brown basally; apices of tibiae and tarsi sometimes infuscate. Metasoma more or less uniformly brown or dark brown in paratypes, or extensively yellow ventrally and posteriorly in holotype (Fig. 27).

**Male.** Unknown.

#### Variation.

All paratypes are smaller (body length ca. 2.5 mm, fore wing length ca. 2.2 mm), paler and with weaker punctures than the holotype. Foveate groove of mesopleuron in paratypes is usually narrow and more or less straight. Shape and length of basal area of propodeum is very variable: 2.0 to 4.0 × as long as broad, and 0.4 to 0.8 × as long as apical area.

#### Etymology.

The species is named after the type locality, [State of] Oaxaca.

#### Material examined.

***Holotype*** female (UNAM), Mexico, Oaxaca, Santiago Comaltepec, 17.58429N, 96.49398W, 2332 m, 6.VI.2009, coll. H. Clebsch & A. Zaldívar.

***Paratypes*.** 7 females (3 in UNAM, 2 in UAT, 2 in ZISP), Mexico, Oaxaca, Santiago Comaltepec, 17.58424N, 96.49428W, 2427 m, Malaise trap, 12–20.VI.2007, coll. H. Clebsch.

#### Distribution.

Mexico (Oaxaca).

## Supplementary Material

XML Treatment for Gelanes

XML Treatment for Gelanes contrerasi

XML Treatment for Meggoleus

XML Treatment for Meggoleus hidalgoensis

XML Treatment for Meggoleus whartoni

XML Treatment for Phradis

XML Treatment for Phradis belovi

XML Treatment for Stethantyx

XML Treatment for Stethantyx covida

XML Treatment for Stethantyx oaxacana
